# Intracranial Stenting: Is It Still an Option for Treatment of Patients With Intracranial Atherosclerosis?

**DOI:** 10.3389/fneur.2019.01248

**Published:** 2019-11-22

**Authors:** Ahmed Abualhasan, Foad Abd-Allah, Guglielmo Pero, Khaled Sobh, Ossama Mansour, Omar El-Serafy, Edoardo Boccardi

**Affiliations:** ^1^Department of Neurology, Faculty of Medicine, Cairo University, Cairo, Egypt; ^2^Department of Neuroradiology, Ospedale Niguarda Ca' Granda, Milan, Italy; ^3^Department of Neurology, Faculty of Medicine, Al-Azhar University, Cairo, Egypt; ^4^Department of Neurology, Faculty of Medicine, Alexandria University, Alexandria, Egypt

**Keywords:** intracranial atherosclerosis, medical therapy, endovascular treatment, stroke, intracranial stenting, angioplasty

## Abstract

Intracranial atherosclerotic disease (ICAD) is considered a major cause of recurrent cerebrovascular events. ICAD continues to be a disease without an effective method of reducing the risk of recurrent stroke and death, even with aggressive, highly monitored medical treatment. We reviewed data from three randomized controlled studies that published data comparing intracranial stenting vs. medical treatment for symptomatic severe-ICAD. Ethnic, demographic, clinical, and procedural differences were observed among the data from these trials that might influence their results. Future research should aim at establishing refined selection criteria that can identify high-risk ICAD patients who may benefit from intracranial stenting.

## Introduction

Intracranial atherosclerotic disease (ICAD) is considered a major cause of recurrent stroke and transient ischemic attacks (TIAs) (1). The optimal treatment for ICAD is a crucial issue in Stroke Medicine. Data from recent trials demonstrated that aggressive medical treatment and lifestyle modifications are better than endovascular treatment for stroke prevention in high-risk patients with ICAD (2, 3). However, the annual stroke risk in patients with intracranial atherosclerosis is still high even with aggressive, highly monitored medical management. Questions remain as to what Stroke physicians should do, if patients suffer ischaemic events in spite of optimal medical treatment? Is endovascular therapy a viable treatment option for a certain subgroup of ICAD patients who are not responding to optimal medical therapy?

## Methods

We searched PubMed and Cochrane Library for Randomized Controlled Trials (RCTs) comparing efficacy and safety of medical treatment vs. intracranial stenting as treatment options for intracranial arterial stenosis. The key words included “intracranial atherosclerosis,” “stroke,” “medical treatment,” “intracranial stenting,” and “randomized controlled trial.” Three main trials were found and included for data extraction. We reviewed the data from the three trials including: patient and procedural characteristics, and outcome parameters (Table 1).

**Table 1:** Comparison between three large randomized trials for management of ICAD.

**Trial**	**SAMMPRIS**		**China RCT for MCA stenosis**		**VISSIT**	
	**Medical group (*n* = 227)**	**Endovascular group (*n* = 224)**	**Medical group (*n* = 34)**	**Endovascular group (*n* = 36)**	**Medical group (*n* = 53)**	**Endovascular group (*n* = 58)**
Study design	Randomized (1:1) multicenter clinical trial		Randomized (1:1) single-center clinical trial		Randomized (1:1) multicenter clinical trial	
No. of Centers	50		1		27	
Interventions	medical therapy	Self-expanding stent	medical therapy	Drug Eluting stent (18) Self-expanding stent (7) Angioplasty (4)	medical therapy	Balloon mounted stent
No. of Patients	451		70		112	
	227	224	34	36	53	59
Age years (SD)	59.5 (11.8)	61.0 (10.7)	49.18 (9.29)	53.42 (13.55)	61.8 (12.82)	61.8 (12.28)
Male sex no. (%)	145 (63.9)	127 (56.7)	25 (73.5)	24 (66.7)	32 (60.4)	41 (70.7)
Race no. (%)	Black 50 (22.0) White 161(70.9) Other 16 (7.0)	Black 55 (24.6) White 160 (71.4) Other 9 (4.0)	Chinese population		White 38 (71.7) Asian 7 (13.2) Black or African American 5 (9.4) Hispanic or Latino 2 (3.8)	White 42 (72.4) Asian 7 (12.2) Black or African American 4 (6.9) Hispanic or Latino 5 (8.6)
Hypertension no. (%)	203 (89.4)	201 (89.7)	15 (44.1)	23 (63.9)	43 (81.1)	49 (84.5)
Diabetes no. (%)	103 (45.4)	106 (47.3)	5 (14.7)	8 (22.2)	20 (37.7)	25 (43.1)
Dyslipidemia no. (%)	203 (89.4)	194 (86.6)	13 (38.2)	10 (27.8)	32 (60.4)	29 (50.0)
Smoking no (%)	Current 69(30.4) Former 80(35.2) Never 78(34.4)	Current 54(24.2) Former 79(35.4) Never 90 (40.4)	Current 19(55.9)	Current 21 (58.3)	Current 12 (22.6) Former 17 (32.1) Never 24 (45.3)	Current 11 (19.0) Former 22 (37.9) Never 25 (43.1)
Coronary artery disease no (%)	59 (26.0)	47 (21.0)	5 (14.7)	3 (9.1)	12 (22.6)	10 (17.2)
Symptomatic qualifying artery	Internal carotid Middle cerebral Vertebral Basilar		Middle cerebral		Internal carotid Middle cerebral Vertebral Basilar	
Arterial stenosis, mean (SD), %	81 (7)	80 (7)	85 (6.74)	83.9 (8.13)	80.4 (7.5)	78.9 (7.3)
Qualifying event no. (%)	Stroke 152 (67.0) TIA 75 (33.0)	Stroke 142 (63.4) TIA 82 (36.6)	Stroke 8 (23.5) TIA 26(76.5)	Stroke 7 (19.4) TIA 29(80.6)	Stroke 34 (64.2) TIA 22 (41.5)	Stroke 36 (62.1) TIA 24 (41.4)
Time from qualifying event to randomization, Median (days)	7	7	187.26	261.95	15	9
Stroke related death at 30 days no. (%)	0 (0.0)	5(2.2)	0 (0.0)	0 (0.0)	0 (0.0)	3 (5.2)
*P*-value	*P* = 0.02		*P* = 1		*P* = 0.25	
Any ischaemic stroke at 30 days no. (%)	12 (5.3)	23 (10.3)	0 (.0)	1 (2.8)	3 (5.7)	10 (17.2)
*P*-value	*P* = 0.04		*P* = 0.33		*P* = 0.08	
Haemorrhagic stroke at 30 days no. (%)	0 (.0)	10 (4.5)	0 (.0)	0 (.0)	0 (.0)	5 (8.6)
*P*-value	*P* = 0.04		*P* = 1		*P* = 0.06	
Target territory related stroke at 12 month no. (%)	23 (10.1)	36 (16.1)	1 (2.9)	1 (2.8)	5 (9.4)	20 (34.5)
*P*-value	*P* = 0.06		*P* = 0.86		*P* = 0.003	

## Trials Overview

The SAMMPRIS (2) and VISSIT (3) trials were initiated to compare aggressive medical treatment vs. intracranial stenting in patients with symptomatic, severe ICAD. Both trials concluded that medical therapy was better than intracranial stenting. In contrary, a single-center randomized controlled trial in China, that randomized patients with stroke or TIA attributable to significant Middle Cerebral Artery (MCA) stenosis, to either endovascular or aggressive medical treatment concluded that endovascular treatment could be safe and efficient treatment modality for carefully selected patients with MCA stenosis (4). Sample sizes were estimated in each trial according to power analysis. The intent of the intervention was to prevent stroke or death within 30 days after enrollment or any stroke, death in the territory of the symptomatic intracranial artery beyond 30 days through 12 months. Enrollment was discontinued in SAMMPRIS and VISSIT trials after negative results and a higher rate of stroke and deaths in the stenting group (2, 3). The technical success rate was 92.9% in the endovascular arm of SAMMPRIS, and 54% in the endovascular arm of VISSIT, and 100% in the endovascular arm of China RCT. The rate of adverse events was significantly higher among stent group compared to optimal medical therapy group in SAMMPRIS and VISSIT trials. In China RCT, there was similar adverse events rate in the optimal medical therapy and stent groups.

## Differences in Patient Selection and Procedural Characteristics

Post-stenting adverse events in the endovascular arm of China RCT were considerably lower compared to the endovascular arm of both SAMMPRIS and VISSIT trials (2–4). The mechanisms of post-stenting adverse events in ICAD patients are usually uncertain (5). Presumed mechanisms include perforator occlusion, distal embolization, wire perforation, cerebral hyperperfusion, or in-stent thrombosis. Some post-procedural adverse events might be related to patient's non-adherence to antiplatelet therapy, risk factor control, or even follow-up visits (6).

Many reasons may contribute to better outcome and less adverse events after intracranial stenting in China RCT. First, the operators were highly experienced in a single center having operated more than 500 ICAD interventions in the 5 years before enrollment. This might guarantee technical success and safe outcome of procedures and maintain the continuity of operators' experience which is critical in reducing periprocedural complications and adverse events (7, 8). In SAMMPRIS, of the 224 patients enrolled, 208 underwent stent placement in 50 participating sites in the United States over 29 months with an average of <2 ICAD interventions at each site per year. This may indicate that SAMMPRIS operators may not have had enough experience with ICAD interventions. Secondly, Chinese neurointerventionists were free in choosing between different endovascular modalities with options of a balloon-expandable stent or self-expanding stent placement based on lesion morphology, vessels tortuosity and the operators' own judgment. Generally, using balloon-mounted stents or primary balloon angioplasty usually do not require over wire catheter exchange with low risk of wire perforation and subsequent haemorrhagic complications (9). Thirdly, the mean age in the Chinese stenting group was 53 years, younger than the VISSIT and SAMMPRIS trials, suggesting easier vascular access for stenting and less procedural complications (10, 11). Additionally, the study was conducted only on Chinese patients excluding diverse ethnic groups with varied stroke pathologies and responses to treatment (12).

In the China RCT, the study population showed less prevalence of stroke risk factors, than the SAMMPRIS and VISSIT trials, e.g., HTN, DM, dyslipidaemia, and coronary artery disease which are associated with increased risk of procedural complications (13). SAMMPRIS trial received criticism for its patient selection and failure to differentiate the most likely stroke mechanism (perforator-occlusion, hypoperfusion, and embolic stroke). SAMMPRIS did not mention inclusion of ICAD patients with hypoperfusion in the territory supplied by the target vessel. Those patients might be more prone to benefit from endovascular revascularization (14). China RCT included only proximal MCA stenosis. Basilar artery stenosis, which may carry a higher periprocedural risk for endovascular treatment was included in the SAMMPRIS and VISSIT trials (8, 13, 15). Finally, most of qualifying events in China RCT were TIAs and time from qualifying events to randomization was longer than the time interval in SAMMPRIS and VISSIT. Recent stroke may be a major risk factor for periprocedural adverse events, which could result from plaque instability (8, 16, 17). The long interval between qualifying event and procedure may allow for stabilization of the atherosclerotic plaque via medical treatment, thus reducing the thromboembolic events in the periprocedural period (8).

## Current Status and Future Directions

Current Stroke guidelines do not recommend endovascular treatment as an initial modality (18). Even for ICAD patients with recurrent stroke or TIAs despite aggressive medical treatment, the usefulness of endovascular treatment is considered unknown and investigational. However, the recently published results of Wingspan stEnt system post-mArket surVEillance (WEAVE) trial showed extremely low periprocedural stroke and death rate (2.6%) with Wingspan stent use (19). The trial data suggest that proper patient selection and operator experience may maximize benefit from endovascular treatment with the Wingspan Stent System. Additionally, China Angioplasty and Stenting for Symptomatic Intracranial Severe Stenosis (CASSISS) trial is an ongoing, multicentre RCT with refined inclusion criteria aiming at determining whether intracranial stenting was superior to aggressive medical treatment for preventing recurrent cerebrovascular events in patients with severe symptomatic ICAD (20). The participating centers in CASSISS trial had to prove an annual volume of more than 30 ICAD patients treated with intracranial stenting sustained over the past 3 years. In CASSISS, patients with perforator ischaemia alone without distal hypoperfusion or artery-to-artery embolism would not be included in the study. Also, pre-procedural perfusion imaging would be used to evaluate for cerebral hypoperfusion. Additionally, time from qualifying event to procedure should not be <3 weeks. Another multicentre RCT is currently underway to compare intracranial stenting with aggressive medical therapy in patients with severe symptomatic ICAD and cerebral hypoperfusion (21). Cerebral hypoperfusion would be assessed initially with transcranial Doppler measuring cerebral blood flow velocity and would be confirmed using Magnetic Resonance perfusion imaging. The final results may clarify the potential role of intracranial stenting in management of ICAD.

## Conclusion

Endovascular treatment of ICAD remains a feasible viable option, but its efficacy in preventing recurrent cerebrovascular events still needs validation. Careful patient selection and technical advancement of the devices utilized may improve the long-term clinical outcomes of intracranial stenting. Future large randomized controlled trials with refined selection criteria are warranted.

## Author Contributions

AA, FA-A, and GP drafted the article. All authors were involved in data acquisition, critical analysis, organization, engaged in the manuscript review, manuscript editing, and final approval of the manuscript.

### Conflict of Interest

The authors declare that the research was conducted in the absence of any commercial or financial relationships that could be construed as a potential conflict of interest.
